# Biomechanical Property of a Newly Designed Assembly Locking Compression Plate: Three-Dimensional Finite Element Analysis

**DOI:** 10.1155/2017/8590251

**Published:** 2017-06-18

**Authors:** Jiang-Jun Zhou, Min Zhao, Da Liu, Hai-Ying Liu, Cheng-Fei Du

**Affiliations:** ^1^Department of Orthopedics, The 184th Hospital of PLA, Spinal Surgery Treatment Center of Nanjing Military Region, Yingtan, Jiangxi Province 335000, China; ^2^Department of Orthopedics, General Hospital of Chengdu Command, Chengdu, Sichuan Province 610083, China; ^3^Tianjin Key Laboratory for Advanced Mechatronic System Design and Intelligent Control, Tianjin University of Technology, Tianjin 300384, China

## Abstract

In this study, we developed and validated a refined three-dimensional finite element model of middle femoral comminuted fracture to compare the biomechanical stability after two kinds of plate fixation: a newly designed assembly locking compression plate (NALCP) and a locking compression plate (LCP). CT data of a male volunteer was converted to middle femoral comminuted fracture finite element analysis model. The fracture was fixated by NALCP and LCP. Stress distributions were observed. Under slow walking load and torsion load, the stress distribution tendency of the two plates was roughly uniform. The anterolateral femur was the tension stress area, and the bone block shifted toward the anterolateral femur. Maximum stress was found on the lateral border of the number 5 countersink of the plate. Under a slow walking load, the NALCP maximum stress was 2.160*e*+03 MPa and the LCP was 8.561*e*+02 MPa. Under torsion load, the NALCP maximum stress was 2.260*e*+03 MPa and the LCP was 6.813*e*+02 MPa. Based on those results of finite element analysis, the NALCP can provide adequate mechanical stability for comminuted fractures, which would help fixate the bone block and promote bone healing.

## 1. Introduction

Less invasive stabilization systems and locking compression plates (LCP) have been widely used in clinical practice [1–3] and provide new options and challenges for modern fracture surgery. However, the plates and screws cannot completely solve all of the problems encountered. Fracture healing requires a relatively stable environment, accurate anatomical reduction, and reliable internal fixation—each of which can shorten the healing time [4]. A long distance between fracture fragments and the trunk indicates the probability of poor healing [5]. For severe comminuted fracture of the long bone shaft, conventional fixation methods using tensile screws or wires would result in poor stability and could destroy the periosteal blood supply. Park et al. [6] and Yang et al. [7] found that there was a higher failure rate in the treatment of nonisthmal femoral shaft nonunions with intramedullary nails. They considered that the main reason of the failure was mechanical instability.

Our research group has developed a new design assembly locking compression plate (NALCP) under Chinese patent (Patent number ZL201220339335.2). This plate is made of Ti-6Al-4V and it has the same materials as the LCP. The main locking compression plate is equipped with runner plates on both sides that can move with the bone blocks and fix bone blocks using universal locking screws. A runner plate is placed on the main locking compression plate, thus making bone blocks integrate with the bone shaft and achieving better stability based on the screw-bone plate angular stability and plate-plate integrity. The aim of this study is to compare and analyze the mechanical properties of NALCP and LCP in the treatment of femoral shaft wedge comminuted fractures (AO classification type 32-C2.1) in the condition of slow walking loading. To accomplish it, the finite element analysis was used.

## 2. Materials and Methods

### 2.1. Design of Runner Plate

**Figure 1:**
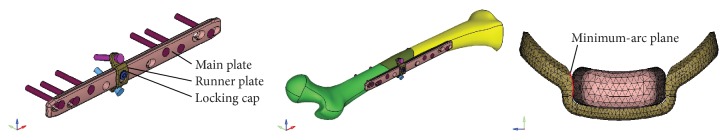
Runner plate integrates with the main plate through the locking female screws. Newly designed assembly locking compression plate (NALCP) fixated in long-bone comminuted fractures. Minimum-arc plane between the inner medial surface of the runner plate and the lateral surface of the main plate.

**Figure 2:**
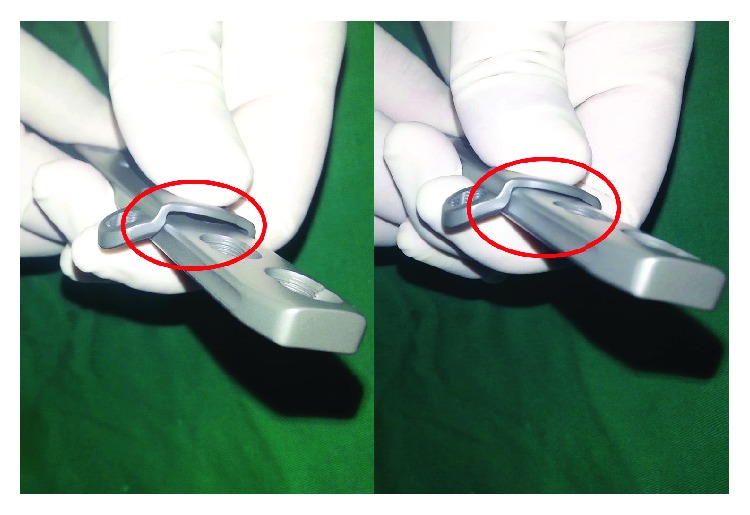
Runner plate can be disassembled from the main plate regardless of its shape: red ellipse circle.

To achieve stability between the main plate and the runner plate, we connected the two plates using locking femoral screws. We also inserted universal locking screws through the runner plate to fix the bone block in a “crossover” manner (Figure 1). The inner medial surface of the runner plate was close to the lateral surface of the main plate, forming a minimum-arc plane. It can be disassembled from the main plate during surgery regardless of its shape (Figure 2).

### 2.2. Establishment of a Finite Element Model for Femoral Fixation

#### 2.2.1. Normal Femoral Geometrical Model

A normal healthy volunteer (age 30 years, height 170 cm, weight 70 kg) underwent computed tomography (CT) scanning of the right lower extremity at a slice thickness of 0.625 mm. The scanned images were imported into medical image processing software Mimics 10.0 (Materialise Technologies, Leuven, Belgium) to segment the skeletal information of femur and then construct the geometry model of femoral in a reverse engineering software (Geomagic 9.0, Geomagic Inc. Morrisville, USA). Finally, the model of femur was converted into nonuniform rational B-spline (NURBS) surface as format of iges.

#### 2.2.2. Femoral Fixation Geometrical Model

**Figure 3:**
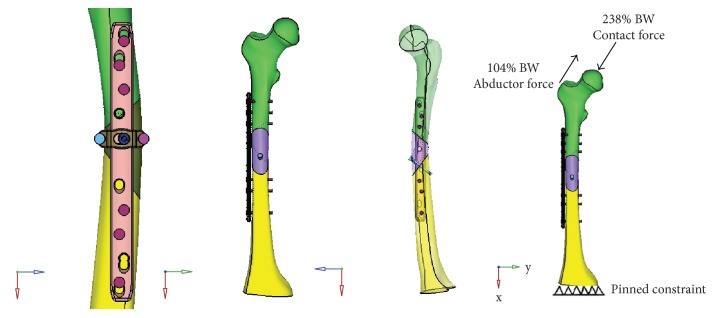
NALCP fixation, LCP fixation, and load imposed on the femur to simulate the slow walking state.

The geometrical model of wedge-fractured femoral was established in the finite element preprocessing software HyperMesh 11.0 (Altair Engineering Corp., Michigan, USA) and then was meshed on the same software platform. The bone fracture gap was set as 0.1 mm. The screw-plate fixation system model was developed by using SolidWorks CAD software (Dassault System SolidWorks Corp., Waltham, USA) (Figure 3). The NALCP main plate and runner plate were then assembled to match the bone shape, and bone blocks were fixed to the runner plate with two screws. For the LCP, bone blocks were fixed with an upwardly inclined screw on the main plate. The two fixation systems were introduced into HyperMesh software to simulate the assembly of fixation systems and femoral fracture models.

#### 2.2.3. Finite Element Model of Femoral Fixation

The fixed femoral geometric entity models were meshed into finite element model in HyperMesh. The screw model was made up of hexahedrons, and the remaining models were formed as tetrahedrons. The overall size of the element was 3 mm [8, 9]. The greatest curvature in the model was treated with Variable Grid Density Biased Sampling technology, and the grid density within the model was coarsened [10, 11]. Some important locations, such as contacts and constraints, were artificially divided to improve calculation accuracy. Finally, two femoral fixation models were established. Their types and quantity of units are shown in Table 1.

### 2.3. Finite Element Analysis

#### 2.3.1. Material Allocation

All models were simulated using homogeneous isotropic linear material. The elastic modulus of the plate was defined as 1.10*e*+05 MPa. The properties of the models' materials after meshing are shown in Table 1.

#### 2.3.2. Model Validation

To confirm the reliability of the results of finite element analysis, we selected complete skeleton model before plate fixation. The load was given in accordance with Wang et al.'s study [12]. Strain of the node at the corresponding position was measured with eight strain gauges.

#### 2.3.3. Definitions of Contact, Constraint, Load, and Boundary Conditions

For simulation of a slow walking load, the frictional contact is defined as contact between the main plate and the runner plate on the fracture surface. The fracture surface friction coefficient is 0.45, and the friction coefficient between the plates is 0.2. The femoral load in single legs during walking was simulated, and the load was given on the surface of the femoral head and the greater trochanter in a point-coupling manner to simulate the acetabular contact force and muscle abduction force. Six random degrees on the distal femur were constrained. The loading direction and size are shown in Figure 3 [13]. A static analysis step was defined in the HyperMesh software with the interface of a finite element solver (Abaqus6.11, Simula Corporation Pennsylvania, USA), and the interaction set, load, and boundary conditions were also added.

#### 2.3.4. Finite Element Method

Two fracture models with preset conditions were saved in .inp format and input into Abaqus for direct discrimination. The discriminating process takes 2 minutes, followed by postprocessing in Abaqus software.

#### 2.3.5. Outcome Measures

The stress distributions of the plates and bone blocks were identified. The bone blocks were detected at the anterior, posterior, lateral, and medial borders of the contact plane at the distal and proximal fracture lines. The values were then averaged.

## 3. Results

### 3.1. Results of Model Validation

**Figure 4:**
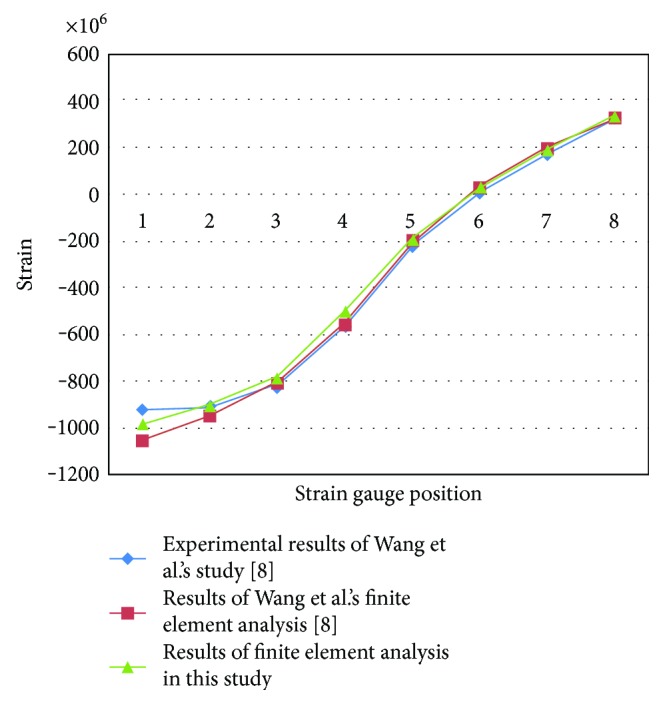
Experimental verification of complete skeleton finite element model. Results of our finite element analysis were consistent with Wang et al.'s results.

Results of finite element model validation of this study was consistent with mechanical test and finite element model results of Wang et al.'s study [12] (Figure 4). We concluded that the establishment of finite element model is credible.

The stress distribution tendencies of the two plates (NALCP and LCP) under two loading conditions were roughly uniform. No concentrated area of stress was found. The anterolateral femur was the area of greatest tension stress, and the bone block shifted toward the same area.

### 3.2. Simulation of Slow Walking Load

**Figure 5:**
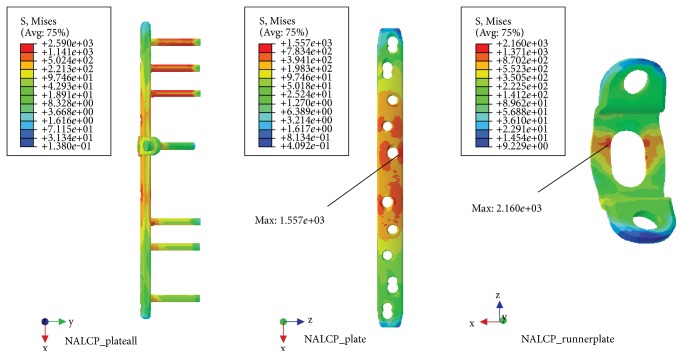
NALCP stress nephogram of the slow walking load.

**Figure 6:**
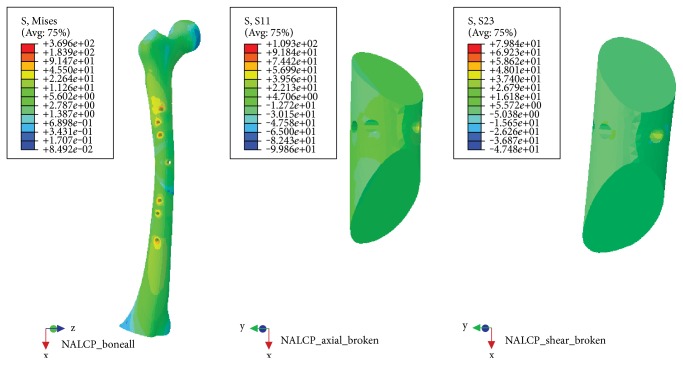
NALCP stress nephogram and bone block axial stress nephogram of the slow walking load.

With the NALCP, maximum stress was situated at the lateral border of the number 5 countersink of the plate (between the runner plate and the main plate locking screw). Maximum stress on the runner plate was located between the locking screws and the runner plate contact area (Table 2, Figure 5). In the skeletal model, maximum stress was distributed at the countersink of the runner plate (Table 3, Figure 6). Under the condition of the slow walking load, the axial maximum stress was 1.834*e*+00 MPa and tensional shear stress was 3.488*e*+00 MPa at the fracture plane.

**Figure 7:**
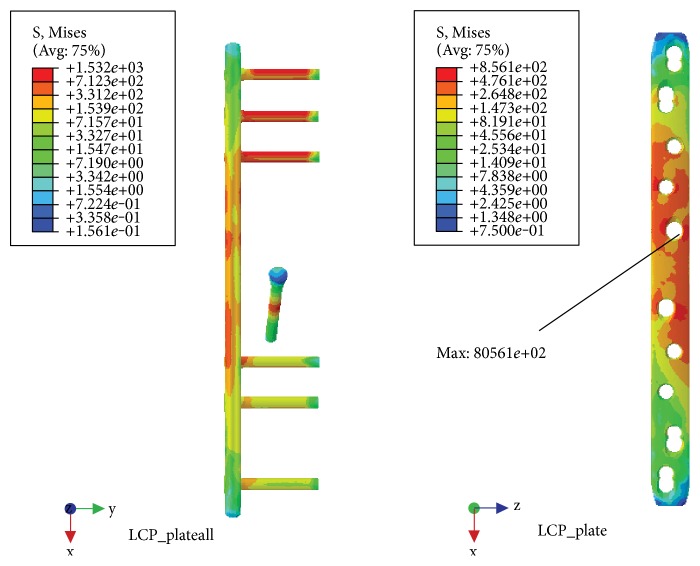
LCP stress nephogram of the slow walking load.

**Figure 8:**
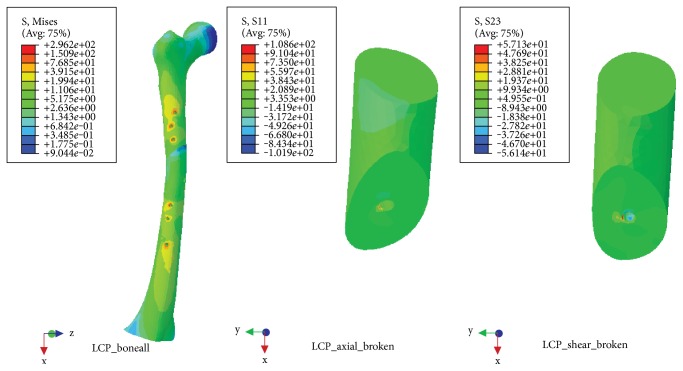
LCP stress nephogram and bone block axial stress nephogram of the slow walking load.

The LCP maximum stress was also located in the lateral border of the number 5 countersink of the proximal plate (Table 2, Figure 7) and showed stress distribution similar to that with the NALCP. Maximum stress in the skeletal model was found in the bone block countersink (Table 3, Figure 8). Under the condition of the slow walking load, the axial maximum stress was 5.858*e*+01 MPa and tensional shear stress was 4.058*e*+00 MPa at the fracture plane.

### 3.3. Torsional Load

**Figure 9:**
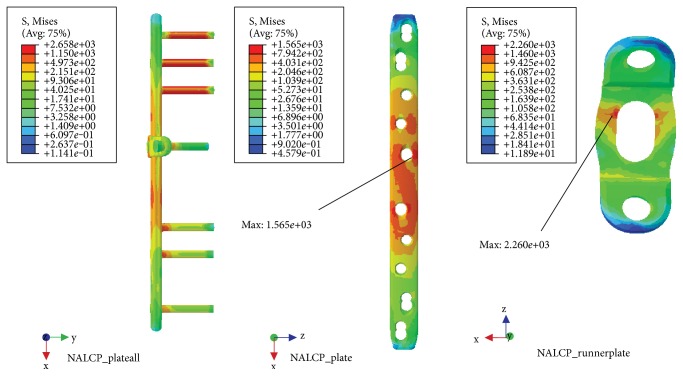
NALCP stress nephogram of the torsional load.

**Figure 10:**
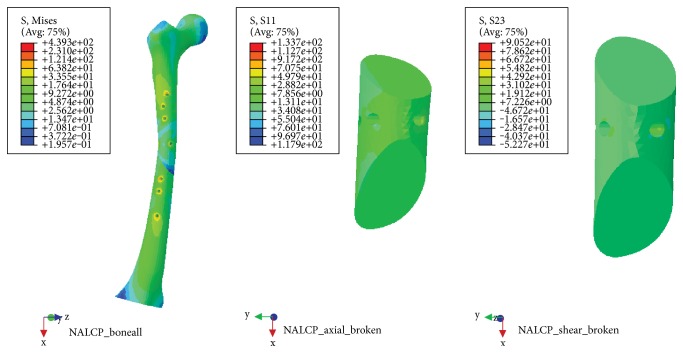
NALCP stress and axial stress nephograms of the torsional load.

NALCP maximum stress was situated in the lateral border of the number 5 countersink of the proximal plate (between the runner plate and the main plate locking screw). Maximum stress on the runner plate was located between the area of contact of the locking screws and runner plate (Table 4, Figure 9). Maximum stress in the skeletal model was distributed at the countersink of the runner plate (Table 5, Figure 10). Under torsional loads, the axial maximum stress was 1.923*e*+00 MPa and tensional shear stress was 3.604*e*+00 MPa at the fracture plane.

**Figure 11:**
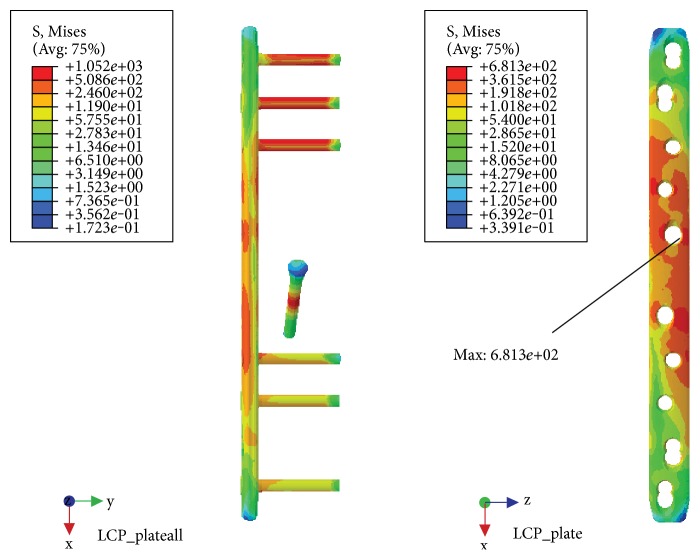
LCP stress nephogram of the torsional load.

**Figure 12:**
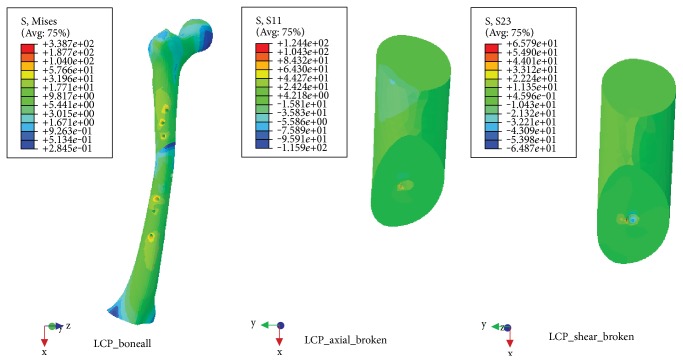
LCP stress and axial stress nephograms of the torsional load.

The LCP maximum stress was also located in the lateral border of the number 5 countersink of the proximal plate (Table 4, Figure 11) and showed stress distribution similar to that seen with NALCP. Maximum stress on the skeletal model was found in the number 1 countersink of the proximal model (Table 5, Figure 12). Under torsional load, the axial maximum stress was 6.660*e*+01 MPa and tensional shear stress was 3.376*e*+01 MPa at the fracture plane.

## 4. Discussion

Modern high-energy trauma often leads to severely comminuted limb fractures. According to the AO classification, type B and C fractures are common. The currently used fixation methods include plate-screw fixation and intramedullary nail fixation. LCP fixation requires at least four holes to reduce stress concentration and avoid plate breakage [14]. Although interlocking intramedullary nails in the treatment of comminuted fractures provide central fixation, high strength, good stability, and small stress concentration as detected by finite element analysis [15], they often induce bone nonunion and intramedullary nail rupture, especially during early weight-bearing activities [4]. With these two fixation methods, bone blocks are often fixed with a single tensile screw or a wire ring. However, fixation is poor and frequently leads to bone displacement.

Previous animal experiments [5] showed that the distance from free bone reflects the apparent impact on fracture healing. Under physiological conditions, when stress is greater than optimal, bone formation dominates in bone metabolism. In contrast, when stress is less than optimal, bone resorption dominates [16]. As for comminuted fractures, especially in weight-bearing bone, axial stress easily leads to a transversal shift of bone blocks [17]. Some authors [18] found that intraoperative unstable rotation and poor contact between bone blocks during treatment of femoral nonunion using intramedullary nails was alleviated after bone grafting and insertion of a steel plate to increase local stability.

Therefore, we designed an arc-shaped surface of the main plate and the runner plate. This innovation is conducive to convenient assembly and disassembly of the runner plate and does not affect the structure of the steel plate. This “micro-arc” design allows arbitrary use of a steel plate during surgery according to the bone blocks. It also increases the flexibility of its use. To enhance fixation, we adopted the use of the locking female screw to lock the runner plate and the main plate. Universal locking screws on both sides of the runner plate can achieve “crossover” triangular fixation of bone blocks. Hence, bone blocks were integrated with the fracture shaft through the runner plate and main plate, achieving strong fixation depending on the screw-plate angular stability.

Finite element analysis has been applied extensively in orthopedic biomechanical analyses and for testing new materials [19–22]. Previous studies focused only on stress distribution of the steel plate and skeleton, leaving stress distribution on the contact surface of the fracture unclear [23, 24]. Also, if only stable fixation was performed after fracture surgery without axial stress, bone callus would grow slowly. Sufficient axial pressure and tension force stimulation and may accelerate the formation of callus at the fracture area [24, 25]. Therefore, under a slow walking load, axial stress of the bone block's contact surface in the finite element analysis can directly reflect postoperative stress stimulation in bone blocks. It is also an indicator of stability of the bone fracture stump after new internal fixation.

A normal healthy volunteer underwent computed tomography (CT) scanning, and the scanned images were imported into several medical image processing software to establish a finite element model. By experimental verification, the results of our finite element analysis were consistent with Wang et al.'s results [12]. Finite element analysis of stress distribution nephogram showed that the stress distribution for the NALCP and LCP systems were similar under slow walking loads. Also, the distance from the bone fracture line to the main plate screws was the high-stress area with both systems, showing uniform stress distribution. Both NALCP and LCP maximum stress was located at the lateral edge of the number 5 countersink of the proximal plate. The maximum stress of the LCP-combined screw fixation was significantly lower than that of the NALCP. The LCP maximum stress was about 54.98% that of NALCP maximum stress. LCP axial stress was 31.94 times that of NALCP axial stress, and the tension shear stress was 1.16 times that of NALCP shear stress. Under torsional loads, NALCP and LCP showed similar stress distributions, and the maximum stress was concentrated at the lateral edge of the number 5 countersink of the proximal plate. The LCP plate maximum stress was 43.53% that of the NALCP plate, the axial stress was 34.63 times that of NALCP axial stress, and the tension shear stress was 9.37 times that of NALCP shear stress. In summary, the axial stress and tension shear stress in the NALCP skeletal model were significantly lower than those in the LCP model. Thus, NALCP enhances the stability of bone blocks under stimulation of a slow walking load and a torsional load [17], improving the fixation effect, preventing lateral displacement of the bone block, and promoting bone healing.

Stoffel et al. [3] investigated the distance of the screw to the fracture site in finite element analysis using the LCP. Their results demonstrated that the maximum stress of the plate occurred at the innermost screw hole. When bone fracture gap was 1 mm during simulating simple fracture, with increasing the distance of the screw to the fracture site, equivalent stress of the plate and the innermost screw was reduced. However, when the bone fracture gap was 6 mm during simulating comminuted fracture, the result was the opposite. Dong et al. [26] also found that for simple femoral shaft fracture, maximum stress on the locking plate and screw gradually decreased with outward movement of the innermost screw. For comminuted fracture, the fracture site could not produce effective contact for stress transfer, so the further deformation of the locking plate could not be prevented. Thus, maximum stress on the locking plate and screw increased. Lee et al. [24] confirmed that for comminuted fracture, locking screws as close as practicable to the fracture site resulted in a high stability of the fixation, and stress concentration did not obviously increase. In our result, the maximum stress of NALCP and LCP are all near the fracture site and are consistent with Lee et al.'s [27] and Xiong et al.'s [28] results. When NALCP obtained an effective stability, the stress on the main plate was less compared with the LCP.

In this study, longitudinal force loaded on the femoral head reached 238% of body weight [12], simulating slow walking, which is much higher than the load value used previously [14, 23, 29]. The results showed that the stress of the NALCP and LCP plates exceeded 600 MPa, the yield strength of titanium alloy. Therefore, the patients could not engage in early full weight-bearing walk. The contact area between the locking screw and the runner plate was the high-stress area. Maximum stress under each of the two loads was higher than maximum stress of the main plate, which is the stress concentration point throughout the fixation system. The potential focus of future studies will be how to reduce stress concentration on the runner plate.

## 5. Conclusion

Based on results of finite element analysis, NALCP can provide a strong mechanical stability for comminuted fractures. NALCP is more convenient to fix bone fragments and to promote bone healing compared with the conventional LCP. Nevertheless, because of the lack of biomechanical experiment of solid specimens, our results deserve further investigations.

## Figures and Tables

**Table 1:** Properties of the models' materials and the number of elements.

Model	Elastic modulus (MPa)	Poisson's ratio	Number of elements	Number of nodes
Cortical bone	1.3*e* + 04	0.3	85 583	19 682
Cancellous bone	2.06*e* + 02	0.3	13 426	58 381
NALCP	1.10*e* + 05	0.3	101 993	35 946
LCP	1.10*e* + 05	0.3	84 600	30 377

**Table 2:** Maximum stress of two fixed plate systems.

Fixation system	Main plate (MPa)	Runner plate (MPa)
NALCP	1.557*e*+03	2.160*e*+03
LCP	8.561*e*+02	—

**Table 3:** Stress distribution of the skeletal model.

Fixation system	Maximum stress	Axial maximum stress (MPa)	Tensional shear maximum stress (MPa)
NALCP	3.696*e*+02	1.834*e*+00	3.488*e*+00
LCP	2.962*e*+02	5.858*e*+01	4.058*e*+00

**Table 4:** Maximum stress of two fixation system plates.

Fixation system	Main plate (MPa)	Runner plate (MPa)
NALCP	1.565*e*+03	2.260*e*+03
LCP	6.813*e*+02	—

**Table 5:** Stress distribution of the skeletal model.

Fixation system	Maximum stress (MPa)	Axial maximum stress (MPa)	Tensional shear maximum stress (MPa)
NALCP	4.393*e*+02	1.923*e*+00	3.604*e*+00
LCP	3.387*e*+02	6.660*e*+01	3.376*e*+01
